# Incidence rate of total knee arthroplasties in eleven European countries: Do they reach a plateau?

**DOI:** 10.1371/journal.pone.0312701

**Published:** 2025-01-07

**Authors:** Mathieu Le Stum, Myriam Le Goff-Pronost, Eric Stindel, Guillaume Dardenne

**Affiliations:** 1 Université de Brest, UBO, LATIM, UMR 1101, Brest, France; 2 Institut National de la Santé et de la Recherche Médicale, INSERM, Laboratory for Medical Information Processing (LATIM), UMR1101, Brest, France; 3 Institut Mines-Telecom, IMT Atlantique, LATIM, UMR 1101, M@rsouin, Brest, France; 4 Centre Hospitalo-Universitaire de Brest, CHRU Brest, LATIM, UMR 1101, Brest, France; Lady Reading Hospital, PAKISTAN

## Abstract

**Background:**

From several decades, the evolutions of the Incidence Rate (IR) of Primary Knee Arthroplasties are continuously increasing worldwide and have been widely studied in several countries. Some recent works have highlighted the fact that the IR is following a sigmoid curve composed of an exponential growth followed by a linear phase and finished by a plateau. Our objective is to assess the IR evolution of eleven European countries, representing thus a large proportion of this continent, regarding this sigmoid.

**Methods:**

IRs of primary knee arthroplasties for Austria, Denmark, Finland, France, Germany, Hungary, Italy, Poland, Spain, Sweden, and the United Kingdom between 2005 and 2019 were retrieved from the EUROSTAT database. Several regression models were fitted to each country’s IRs: Poisson, linear, asymptotic, logistic, and Gompertz regression. For each country and each model, the RMSE (Root Mean Square Error) and R^2^ were calculated and used to estimate their position with respect to this sigmoid curve.

**Results:**

The best regression models for knee arthroplasties varied following countries. Logistic and Gompertz regressions had the lowest RMSE and R2 values for Austria, Denmark, Germany, Sweden, and the UK. Hungary, Italy, and Poland favored the Poisson regression model. Finland and Spain presented difficulties in determining the optimal model (linear or Poisson), while France faced challenges in choosing between logistic, Gompertz, and linear regression.

**Conclusion:**

In conclusion, the growth dynamics of IR differ across European countries. Some countries seem to have already reached a plateau and will therefore experience slight growth in the future.

## Introduction

Primary knee arthroplasties are among the most common orthopaedic procedures worldwide. In Europe alone, according to EUROSTAT, there were 754,622 such interventions in 2019, and 680,114 in 2014 in the United States [1]. Over the past few decades, the incidence rate (IR)—that is, the number of procedures per 100,000 people—has increased unevenly across different countries. For instance, in the USA, the IR for primary knee arthroplasties rose by 119% from 2000 to 2014. In England, it is by 100% between 1991 and 2000 [2], while in Sweden it increased by 165% from 1995 to 2013 [3]. Austria also saw a continuous rise in the number of primary knee arthroplasties procedures between 2009 and 2015 [4].

While this overall increase is mainly supported by the natural augmentation of the osteoarthritis incidence which is mainly driven by the combined effect of an ageing population [5, 6] and obesity [7], it is also the result of the extended arthroplasty indications on younger patients, mainly related to the improved surgical techniques and the implant quality [5, 8, 9]. At the same time, faced with rising costs of caring for patients living longer and more actively, health systems will be confronted with sharply increasing expenses. A precise analysis seems imperative to anticipate future management and financial issues [10–18].

Although uneven increases in primary knee arthroplasty IR have been observed in all OECD countries [19, 20], disparities exist between countries and trends projection models. These models, which are mostly constructed based on past data, are often based on exponential or linear growth. However, recent works have questioned the systematic and almost exponential increase of IR and emphasize the fact that a stagnation of the IR is expected, leading to an upper asymptote. In 2014, Nemes et al. first proposed a sigmoid curve model, showing an exponential growth phase followed by a stagnation phase with minimal or no growth, reaching a maximum IR [21]. An extensive analysis of historical data from Sweden demonstrates a gradual slowdown in growth since the 1980s [3], with similar trends seen in Denmark but occurring later, after the 2000s [22]. Studies using the National Inpatient Sample (NIS) database for the United States revealed weak growth from 1993 to 1999, followed by accelerated growth from 2000 to 2012 [1, 23]. Additionally, a slowdown was observed from 2008 to 2014 [1].

Our hypothesis asserts that the growth trajectories of primary knee arthroplasty incidence rates in European countries exhibit distinct patterns with a conclusive trend toward stabilization. This study endeavours to evaluate historical incidence rates to enhance the precision of forecasting potential future trends, to better adapt healthcare systems.

## Materials and methods

### Data

The IR of primary knee arthroplasties, were directly retrieved from the European Union EUROSTAT database (https://ec.europa.eu/eurostat/) between 2005 and 2019 for eleven European countries: Austria, Denmark, Finland, France, Germany, Hungary, Italy, Poland, Spain, Sweden, and United Kingdom (UK). Due to the Brexit, the data for UK from the year 2019 has been extracted from the OECD database (https://stats.oecd.org). A data quality control was conducted between the Eurostat and OECD databases from 2005 to 2018 for UK to assess similarity between both databases. The data were found to be similar. Data for 2020 and 2021 have been excluded of our study because this period was not representative of the natural evolution due to the COVID crisis. Data for 2022 and 2023 are not yet available.

Countries were selected based on the following criteria: (1) representativeness of different European regions, (2) availability of complete data throughout the study period, and (3) previous inclusion in international study for comparative analysis. Data extraction utilized the ICD-9-CM 81.54 (total knee replacement) code, a standard classification maintained and updated by the World Health Organization for international comparisons [19, 20]. The EUROSTAT database, which gathers official data from each national health systems and harmonizes it with the ICD-9-CM classification through quality control, ensured consistent data acquisition across all European countries.

All studies involving human participants adhered to ethical standards set by institutional or national research committees and the 1964 Helsinki Declaration, including subsequent amendments or similar ethical standards.

As previously noted in the literature [4, 24], IR of primary knee arthroplasties have been categorized into three groups: high (above 200), medium (between 100 and 200), and low (below 100) IR. This classification system allows the comparison of the number of arthroplasty procedures performed in different countries, based on the scale of IR.

### Statistics

Historical growth rates across countries were calculated by using the compound annual growth rate (CAGR), which is a geometric progression ratio that estimates the smoothed annual growth rate by considering the values at the beginning and the end of the period under study. The CAGR allows the comparison of growth rates over time [3, 20] as it reduces the impact of short-term fluctuations. In each country, we compared two similar time periods (8 and 7 years, respectively): the period from 2005 to 2012, which includes the economic downturns of 2008–2009 as well as the financial crisis and European debt crisis between 2010 and 2012- [25] and the period from 2013 to 2019 which corresponds to the post-recession years before the crisis of COVID-19. These time periods were selected to align as closely as possible with those previously used in the literature [1, 3, 22, 23, 25–28].

### Models

To analyze the data from a dynamic point of view, three types of regression analysis were considered over the entire period in this study: (1) a Poisson regression analysis assuming exponential growth throughout the time period, (2) a linear regression analysis assuming steady growth over time, and (3), as introduced by Nemes et al [3, 21], a regression framework assuming the existence of an upper asymptote and composed of three competing models: asymptotic, logistic, and Gompertz regressions. Since it is presumed that the population’s growth is not indefinite, the sigmoid curve was subsequently examined.

The sigmoid curve, which characterizes many biological processes of growing population [29], can be divided into three distinct phases which are (1) the lag phase and exponential take off, (2) the linear phase, and (3) the plateau phase [30, 31]. These three steps come one after the other and can define different levels of maturity based on their ability to fit the data. Thus, it is assumed that if the IR evolution of a country aligns closely with a linear or Poisson regression, it suggests constant or exponential growth over time, corresponding to the linear or exponential phase. Conversely, if the IR evolution is closer to an asymptotic regression provided by the three competing models, it suggests that the country is reaching the plateau phase.

Poisson regression model parameters were estimated using the Wald test. The quality of adjustment, including goodness of fit and maximum likelihood, was evaluated using deviance and Pearson chi-square values [21, 32]. Linear regression parameters were estimated using the student test, and assumptions of normality (Shapiro test), absence of heteroscedasticity (Breusch Pagan test), and autocorrelation (Breusch-Godfrey test) were assessed [1]. Asymptotic, logistic, and Gompertz regressions utilized nonlinear least-squares estimation with the Levenberg-Marquardt optimization algorithm to determine the asymptote and parameters [3, 21]. All models were validated with an alpha risk of 5%.

Adjusted R^2^ was calculated for linear regression, while pseudo-R^2^ values were computed for Poisson, asymptotic, logistic, and Gompertz regressions to assess their performance [1].

In order to determine the most appropriate model, we used the Root Mean Square Error (RMSE), which measures the difference between the predicted and observed values. A smaller RMSE value indicates greater accuracy of the model’s predictions.

All statistics were conducted using the R-4.0.2 software.

Ethical approval was not required for the study protocol as publicly available data from EUROSTAT were utilized.

## Results

The countries included in the study had a total population of 401.8 million people in 2019, accounting for 78.3% of the European Union’s population (including United Kingdom). These countries also reported 653,011 primary knee replacements, representing 86.5% of all primary knee replacements in the region.

**Fig 1:**
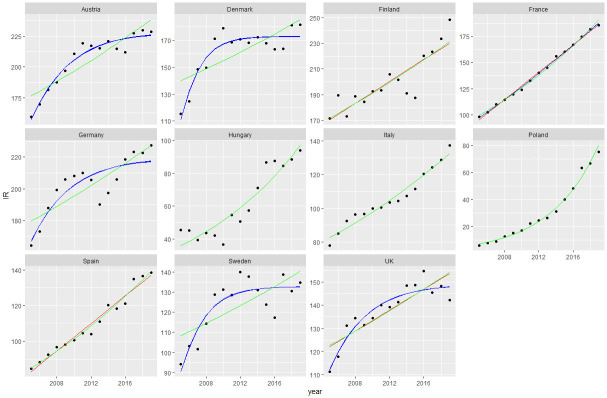
From 2005 to 2019, Incidence Rate (IR) and projections for the knee estimated using the three (asymptotic, logistic and Gompertz) competed models (blue), the Poisson (green) and the Linear (red) models. Only validated models are represented.

The volume of primary knee replacements increased by 60% during the study period, from 390,000 to 653,011. Between 2005 and 2019, the IR increased for all studied countries. In 2019, Austria, Denmark, Finland, and Germany had a high IR, while France, Italy, Spain, Sweden, the United Kingdom, and had a medium IR. Hungary and Poland had a low IR (Fig 1).

The Compound Annual Growth Rate (CAGR) of the IR and volume increased for all the studied countries from 2005 to 2019. However, this growth was not uniform, with Poland experiencing the strongest increase and Austria, Finland, Germany, Sweden, and the United Kingdom showing the lowest growth rates. There is also a notable difference between the two sub-periods. For instance, compared to the 2005–20012 period, Hungary had a considerable increase during 2013–2019, whereas Sweden, the United Kingdom and Austria showed a significant decrease. Some countries slightly moved up (Finland, Italy and Spain) or down (Denmark, France, Germany and Poland) in the second period, while Spain remained stable (Table 1).

**Table 1:** Incidence rate and number of procedure in the 11 European countries selected. CAGR = Compound Annual Growth Rate.

Country	Incidence Rate (IR)				CAGR IR			Number of procedure				CAGR Volume		
	2005	2012	2013	2019	2005–2019	2005–2012	2013–2019	2005	2012	2013	2019	2005–2019	2005–2012	2013–2019
Austria	159.5	217.1	215.3	228.6	2.6%	4.5%	1.0%	13126	18303	18260	20296	3.2%	4.9%	1.8%
Denmark	115.3	170.9	168.5	203.2	4.1%	5.8%	3.2%	6248	9558	9461	11813	4.7%	6.3%	3.8%
Finland	171.6	206.0	201.6	242.1	2.5%	2.6%	3.1%	9003	11154	10963	13369	2.9%	3.1%	3.4%
France	98.4	140.3	145.2	185.0	4.6%	5.2%	4.1%	61986	91830	95525	124648	5.1%	5.8%	4.5%
Germany	164.0	205.5	190.0	227.4	2.4%	3.3%	3.0%	135241	165252	153210	188985	2.4%	2.9%	3.6%
Hungary	45.4	50.7	57.3	93.9	5.3%	1.6%	8.6%	4584	5030	5671	9172	5.1%	1.3%	8.3%
Italy	77.7	103.5	104.4	137.4	4.2%	4.2%	4.7%	45048	61618	62896	82067	4.4%	4.6%	4.5%
Poland	5.7	24.5	26.2	75.1	20.2%	23.2%	19.2%	2164	9326	9975	28508	20.2%	23.2%	19.1%
Spain	84.5	104.0	111.1	138.6	3.6%	3.0%	3.8%	36903	48662	51784	65316	4.2%	4.0%	3.9%
Sweden	94.1	139.9	137.6	134.6	2.6%	5.8%	-0.4%	8495	13314	13211	13833	3.5%	6.6%	0.8%
United Kingdom	111.2	139.2	141.5	142.2	1.8%	3.3%	0.1%	67202	88682	90703	95004	2.5%	4.0%	0.8%
Total	100.0	131.0	130.4	159.8	3.4%	3.9%	3.4%	390000	522729	521659	653011	3.8%	4.3%	3.8%

The IR and regressions for all countries are presented in Fig 1, and the RMSE and R^2^ values are provided in Table 2.

**Table 2:** RMSE and pseudo R^2^ or R^2^ (between brackets) for the primary knee arthroplasties. Boxes are empty (-) for non-validated models.

*Country*	Asymptotic regression	Logistic regression	Gompertz regression	Linear regression	Poisson regression
Austria	5.0 (0.94)	4.9 (0.95)	4.9 (0.95)	-	9.9 (0.78)
Denmark	-	6.6 (0.87)	6.8 (0.87)	-	12.9 (0.52)
Finland	-	-	-	10.7 (0.73)	10.3 (0.76)
France	-	1.4 (0.99)	1.4 (0.99)	1.8 (0.99)	2.2 (0.99)
Germany	8.7 (0.75)	8.9 (0.74)	8.8 (0.75)	-	9.6 (0.69)
Hungary	-	-	-	-	7.2 (0.85)
Italy	-	-	-	-	3.2 (0.96)
Poland	-	-	-	-	2.5 (0.99)
Spain	-	-	-	3.0 (0.96)	2.6 (0.98)
Sweden	-	6.6 (0.77)	6.7 (0.77)	-	10.1 (0.48)
United Kingdom (UK)	3.7 (0.89)	3.8 (0.89)	3.7 (0.89)	5.8 (0.72)	6.1 (0.72)

The smallest RMSE is observed with:

- Asymptotic, logistic and Gompertz for Austria, Germany and UK.
- Logistic and Gompertz regressions for Denmark and Sweden.
- Poisson regression for Hungary, Italy and Poland.
- Either Linear or Poisson regressions for Finland and Spain.
- Logistic, Gompertz, linear regressions for France

Based on the results from this analysis, we can deduce that European countries are situated in different dynamic phases along the sigmoid curve. Thus, for primary knee arthroplasties, Austria, Denmark, Germany, Sweden, and the UK reach the plateau phase since the completed models, such as the asymptotic, logistic, or Gompertz regressions obtained the best RMSE and R^2^. In contrast, since the Poisson regression is better suited for Hungary, Poland and Italy, these countries are in the early stages of the sigmoid.

Results are not so straightforward for some countries which are probably in an intermediate phase because of equivalent RMSE and R^2^ for different models. For knee arthroplasties, Finland and Spain are likely at the end of the exponential phase and entering the linear phase, since both the Poisson and the Linear regression models are the best fitted models to their IR. For France, a similar conclusion can be made, as it appears to have reached the deceleration phase following the exponential and linear phases.

## Discussion

We can observe from this study that significant IR dissimilarities (levels and percentage growth) can be observed between the European countries for primary knee arthroplasties.

However, this analysis alone does not provide a comprehensive understanding of the evolution of IR over the studied period. Therefore, growth curves are valuable tools that can describe how a variable increases over a time interval until it approaches its saturation value [33]. Recently, several studies have questioned the systematic and almost exponential increase of IR observed in the literature, emphasizing the potential for stagnation and reaching an upper asymptote. Based on this assumption, we have assessed the IR evolution of several European countries for knee arthroplasties.

Different regression models, including linear, Poisson, and three competing models (asymptotic, logistic, and Gompertz), have been applied to the available data. These models were analysed for their validity and RMSE to determine the most probable growth model during the 15 years of the study. Based on this analysis, it can be observed that Sweden, UK, Denmark, Germany, and Austria better fit the competing models, reaching their maximum incidence rates. In contrast, Poland, Hungary, and Italy are most suited to an exponential growth model. Three countries are in an intermediate position: Spain and Finland exhibit characteristics between exponential and linear growth, while France shows traits between linear and the competing models. For the former group, this may indicate that they are at the end of their exponential growth and entering the linear phase. For the latter, it may suggest that it is transitioning from the linear to the asymptotic phase.

Published studies for specific countries confirm these observations for primary knee arthroplasties. For instance, previous authors have shown that Sweden [3, 21] and Denmark [22] are experiencing a slowdown in their IR, indicating an entry intothe asymptotic phase, resulting in reaching a maximum IR. In Germany, Worlicek et al. [34] and Pilz et al. [35] have also observed a slight increase, indicating the reach of the plateau. Likewise, in Austria, an asymptotic pattern and continued growth have been observed [4]. For France, the existence of linear or logistic growth, with difficulties to segregate, is shown by detailed analyses of the national database [36, 37]. In the UK, moderate growth was observed in the 1990s, followed by accelerating growth in the first decade of the 2000s [38, 39]. This observed profile therefore seems to be consistent with our positioning on the sigmoid curve.

Although all countries are expected to exhibit asymptotic behavior, the timeframes for reaching this phase vary. The specific factors contributing to this quasi-stabilization of incidence rates (IR) still need to be determined precisely and are likely to be country-specific. Possible factors influencing these patterns are multiple and may include, in addition to commonly accepted causes such as an ageing population and increasing obesity, factors like economic growth, access to healthcare systems, care protocols, demographic changes, or the number of orthopedic surgeons.

Thus, for the countries located in the exponential or the linear phase, three combined factors can explain this situation: (1) the improvement of the capacity to carry out these procedures, such as the development of specialized structures, perioperative care, and medical demographics, as seen for exemple in France or Spain [37, 40, 41]; (2) the growing number of procedures performed in patients aged 64 and below, as observed in all OECD countries [19, 20]; and (3) certain incentive-based policy decisions, as in Finland [42]. On the other hand, for countries that have reached the plateau phase or are near it, as Sweden Denmark or Austria, factors such as healthcare system (reimboursement for all), few hindrances to acessing surgery and an IR for knee replacements that aligns closely with the actual need for joint replacement [3, 4, 21, 22] can explain this situation.

Although we have demonstrated a three-step growth pattern in countries, leading to a theoretical plateau and maximum IR, this same IR varies significantly among different countries. The highest levels are observed in northern and central Europe. Apart from the demographic and medical factors mentioned earlier, this high IR can be partly attributed to an increased rate of surgical interventions, rather than a higher incidence rate of osteoarthritis itself [19, 20]. The preference for surgical treatment over non-surgical options has shifted, allowing for earlier selection of surgical treatment [43]. For primary knee arthroplasties, the IR has been observed to rise faster in patients under 65 years of age in Nordic countries (Denmark, Norway, Finland, and Sweden) and the USA compared to other age groups. This trend can be attributed to a broader range of indications for primary knee arthroplasties, which now includes younger patients [42], in addition to a more active population susceptible to sports-related injuries [43–45], and increased patient demand influenced by direct-to-consumer advertising [43]. It is interesting to note that in OECD countries, there is a positive correlation between healthcare expenditure and the utilization rate of knee arthroplasties. Specifically, it has been observed that the rate of knee replacements increases as economic resources grow, providing better accessibility for younger patients. Consequently, in countries with limited economic resources, priority is given to elderly patients over younger individuals for joint replacement [19, 20]. This ongoing development of surgical care can also contribute to an increase in the number of interventions due to economic pressures. The desire to maintain an active lifestyle and advancements in prosthetic technology may lead patients to take a more proactive approach and express interest in replacement surgery themselves. Additionally, through discussions with their physicians, patients can become more informed about the available opportunities, raising questions about the concept of induced demand [43] as observed in the United States. All these factors can contribute to the potential for overtreatment of osteoarthritis through arthroplasties as seen in Austria or Germany [4, 34].

The main limitation of this study is related to the data provided by EUROSTAT, which are connected to each country’s national accountability system. Therefore, variations may exist between the data officially reported to EUROSTAT and the data utilized in previous studies conducted in each country, particularly in Northern European countries. Despite EUROSTAT’s efforts to gather consistent data from various official sources, coding effects can still occur, as well as biases in the communication of data from individual countries. Additionally, the use of international codes (e.g., ICD-9 classification) was necessary for cross-country comparability, but these codes cannot be further segmented or subsetted in the available international databases.The second limitation is linked to the fact that an analysis by gender or age is not possible with this database. In fact, EUROSTAT database does not provide data stratified by gender and age. This can only be done by an analysis of each national database, that are difficult to access to non nationals. In these national databases, having detailed informations on variable such as Body Mass Index, osteoarthritis, practice of sport or socio-professional category would also be useful to better understand the evolution of each national societies so as to the main national associated factors linked to arthroplasties.The third limitation is due to the COVID pandemic crisis and the lockdown. Therefore, data from the years 2020 and 2021, which do not accurately reflect the usual activity, were excluded from this study. It would be interesting to take them into account in future years in order to determine if they have had an impact on the past dynamics. Three scenarios could emerge from this epidemic. It might have only a temporary effect, such as a brief disruption, without impacting past dynamics. Conversely, these dynamics could be altered. An acceleration could be seen in the following years, as a catch-up effect, followed by a slowdown. Alternatively, a slowdown could occur directly, linked to a change in practices resulting from this epidemic. Finally, the last limitation is that we focused on Europe due to the unavailability of USA data for the period studied in international databases.

## Conclusion

In conclusion, this study emphasizes the diverse dynamics of knee joint trends across European countries. Those at the asymptote can anticipate limited future changes in incidence rates (IR), while countries in exponential or linear phases will likely reach a plateau. Precisely predicting the timing of this plateau requires a comprehensive multi-year analysis.
